# Transport and localization on dendrite-inspired flat band linear photonic lattices

**DOI:** 10.1038/s41598-023-39985-8

**Published:** 2023-08-11

**Authors:** Javier Cubillos Cornejo, Diego Guzmán-Silva, Víctor Hugo Cornejo, Ignacio Bordeu, Rodrigo A. Vicencio

**Affiliations:** 1 Departamento de Física, Facultad de Ciencias Físicas y Matemáticas, Universidad de Chile, Santiago, Chile; 2 Millenium Institute for Research in Optics - MIRO, Santiago, Chile; 3 Neurotechnology Center, Department of Biological Sciences, Columbia University, New York, NY 10027 USA

**Keywords:** Optical physics, Photonic devices, Electronic properties and materials, Permeation and transport, Neural circuits

## Abstract

The capacity of a physical system to transport and localize energy or information is usually linked to its spatial configuration. This is relevant for integration and transmission of signals as performed, for example, by the dendrites of neuronal cells. Inspired by recent works on the organization of spines on the surface of dendrites and how they promote localization or propagation of electrical impulses in neurons, here we propose a linear photonic lattice configuration to study how the geometric features of a dendrite-*inspired* lattice allows for the localization or propagation of light on a completely linear structure. We show that by increasing the compression of the photonic analogue of spines and thus, by increasing the coupling strength of the spines with the main chain (the “photonic dendrite”), *flat band* modes become prevalent in the system, allowing spatial localization in the linear – low energy – regime. Furthermore, we study the inclusion of disorder in the distribution of spines and show that the main features of ordered systems persist due to the robustness of the flat band states. Finally, we discuss if the photonic analog, having evanescent interactions, may provide insight into linear morphological mechanisms at work occurring in some biological systems, where interactions are of electric and biochemical origin.

## Introduction

**Figure 1:**
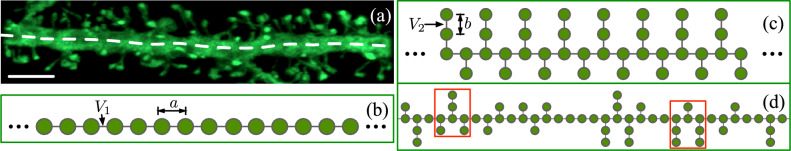
(**a**) Fluorescent image of a dendrite with spines from a pyramidal neuron of human cortex. The dashed line traces the dendrite, and a large number of spines are seen protruding from it in all directions. Scalebar 15 $\mu$m, photo courtesy of Javier DeFelipe, Cajal Institute^32^. (**b**) 1D chain as a photonic dendrite analog. (**c**) [(**d**)] Photonic dendrite with alternated [random] distribution of short and long spines. Red rectangles in (**d**) indicate FB units.

Neurons are responsible for transmitting electrical signals in the brain^1^. A neuron integrates electrical signals via its dendrites [see Fig. 1a], thin ramified cellular protrusions that receive multiple inputs. Through coordinated discharges, dendrites may cause the “firing” of a neuron, whereby an action potential propagates and is transmitted by the axon. Dendrites are populated by surface “spines” [see Fig. 1a], small 0.5–2 $\mu$m long protrusions, that mediate most of the synaptic connections with other neurons^2,3^. Spines are connected to the dendrites by a narrow neck with a high electrical resistance. It has been argued that this allows dendrites to integrate electrical signals from multiple spines and transfer them to the dendrite in a coordinated manner^4,5^. Furthermore, the highly variable morphology of spines and their spatial organization around the dendrites are key to understand basic features of synaptic plasticity^6–8^. Inspired by the spatial organization of spines, and their role in the integration and propagation of electric signals from individual synapses to the neuron – a role that has only recently become accessible by in-vivo techniques^9–11^—we investigate here the transport properties of such configurations in a photonic lattice counterpart.

Researchers from different fields have investigated the possibility of mimicking lattice phenomena on diverse experimental contexts, opening the possibility for exploring transport and localization properties on systems ranging from material science^12^, to mechanics^13^, and photonic setups^14^, among many others. Specifically, photonic lattices have provided the means for visualizing directly, with a simple CCD camera, many theoretical predictions from condensed matter^15^. For example, the study of topological properties^16^ or observing the localized wavefunction in disordered systems^17,18^. For several years, energy localization was a fundamental topic of research^19^, where only recently Flat Band (FB) systems provided a simple and linear solution^20–22^. A FB lattice geometry allows the cancellation of amplitudes at specific connector sites due to a destructive interference process^23,24^, which causes the emergence of localized and perfectly compact (zero tail) wavefunctions that can be combined to form complex coherent patterns^25–28^. Interestingly, FB are not limited to periodic systems only; they can exist as long as a specific FB *unit* is present through the lattice^24^ as, for example, in a fully disordered system^29^.

In this work, we study a photonic lattice whose geometry is inspired by the spatial organization of spines around a dendrite [see Fig. 1a]. The interactions in dendrites are mediated mainly by the transport of ionic charges, yet in optical systems they are mediated by the diffractive interaction (referred as *coupling*) between the optical modes of different lattice sites. Despite these differences, a simple photonic system is useful to gain insight into the role of topology in the transport properties of the system, and might inspire studies in biological systems to explore similar behaviours in the linear regime. In the photonic system, we show that depending on the spatial configuration and coupling of the *photonic* spines with the dendrite, the system may exhibit only extended (propagating) modes or FB states. More specifically, we construct a *photonic* dendrite as a homogeneous one-dimensional (1D) chain of coupled oscillators [as shown in Fig. 1b], which allows the free propagation of a given stimulus, without experiencing any localization feature^22,30^. This system is typically used as an analogue of single continuous waveguides in which a pulse can radiate^31^. In the context of a neuron, this chain mimics a single dendrite that communicates external electrical inputs to the soma, the main body of a neuron^1^. We then consider the *photonic* dendritic spines as perpendicular extensions that protrude from the main chain^29^, forming a fully periodic or random structure as the examples shown in Fig. 1c and d, respectively.

## Photonic dendrite-like lattice model

A photonic lattice^22^ is typically composed of single-mode waveguides with a well defined propagation constant, which defines their evolution on the propagation coordinate *z*. In this sense, a waveguide perfectly mimics an oscillator which evolves in time^19^. The propagation of light on a given photonic lattice [as those sketched in Fig. 1b–d] is described by a tight-binding-like model^19,30,33^ generally written as

1 $$\begin{aligned} -i\frac{du_{\vec {n}}}{dz}=\sum _{\vec {m}\ne \vec {n}}V_{\vec {n},\vec {m}}u_{\vec {m}}\;, \end{aligned}$$

where we have assumed single-mode waveguides only^34^. $u_{\vec {n}}$ describes the light amplitude of the waveguide mode at the $\vec {n}$-th site, while $V_{\vec {n},\vec {m}}$ defines the coupling coefficient in between sites $\vec {n}$ and $\vec {m}$ depending on the lattice geometry (throughout this work, only nearest-neighbor coupling is considered). We identify $V_1$ and $V_2$ as the horizontal and vertical couplings coefficients [see Fig. 1b and c], respectively, and define a *compression parameter* $\delta \equiv V_2/V_1$. Small values of $\delta$ equate to dilated spines (high resistivity and low spine coupling), while larger values of $\delta$ correspond to compressed spines (high spine coupling, small resistivity). The normal modes of model (1) define the linear spectrum and the transport properties of a given lattice^30^; they can be found using a Bloch-like ansatz^15^: $u_{\vec {n}}(z)=A \exp (ik_x a \vec {n})\exp (iE z)$, where *A* is an amplitude at a given site of the unit cell. $k_x$ corresponds to the transversal momentum along the dendrite direction and “*a*” to the dendrite spacing [see Fig. 1b]. *E* defines the propagation constant of the normal modes along *z*. To characterize the effective size of a given profile we use the *participation ratio* defined as $R\equiv (\sum _{\vec {n}} |u_{\vec {n}}|^2)^2/(N\sum _{\vec {n}} |u_{\vec {n}}|^4)$, where *N* is the number of lattice sites. $R\sim 0$ corresponds to a highly localized profile, while $R\sim 1$ to an extended distribution.

Dendrite lattices having a periodic distribution of short or long spines only exhibit transport due to a fully dispersive spectrum (see Section S1 of Supplemental Information). Inspired by the seemingly more complex organization of real dendrites [see Fig. 1a], with spines ranging in sizes and shapes, we question whether a binary spine arrangement could indeed promote the coexistence of extended and localized states. We first focus our attention on a periodic dendrite lattice with alternated short and long spines, as the one sketched in Fig. 1c. This lattice has five sites per unit cell and, therefore, its spectrum is given by:

2 $$\begin{aligned} E(k_x)=0,\ \pm \delta V_1,\ \pm 2V_1\sqrt{\cos ^2 (k_x a)+\delta ^2/2}. \end{aligned}$$

**Figure 2:**
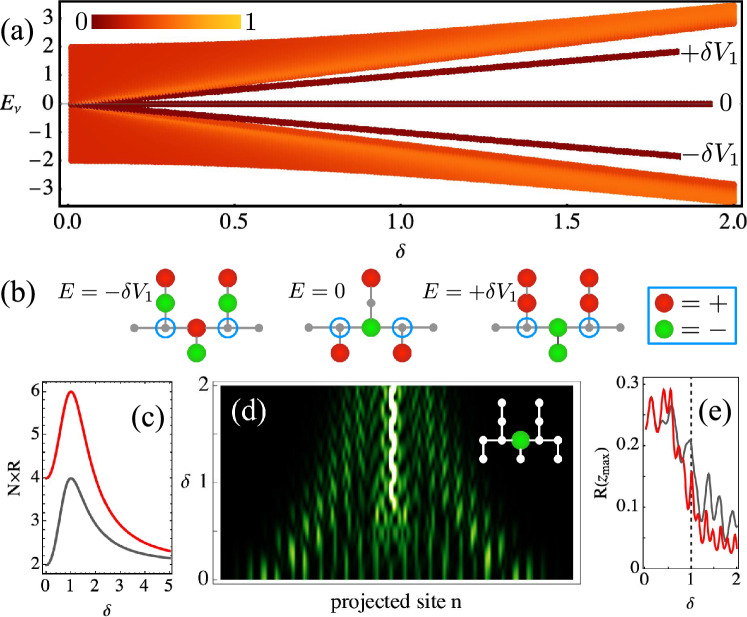
(**a**) Spectrum vs $\delta$ for a dendritic system with alternated spines [see Fig. 1c]. The color bar indicates *R*. (**b**) FB mode profiles for $\delta =1$. (**c**) $N\times R$ vs $\delta$ for FB states at $E=0$ (gray) and $E=\pm V_2$ (red). (**d**) Projected output intensity profiles at $z_{max}=10$ vs $\delta$ (inset: input condition). (**e**) $R(z_{max})$ vs $\delta$ for a dendritic excitation at short (red) and long (gray) spines region.

The first three bands in (2) correspond to the Flat Bands of this lattice system^22^ and, therefore, 3/5 of the spectrum is compact. This implies that the dynamic is strongly determined by the effective size of the FB states, which naturally depends on the ratio $\delta$. For a finite system [see the linear spectrum in Fig. 2a], the extended modes with larger *R* (red-orange colors) form two dispersive bands, while the three FBs emerge at $E=0$ and separate for an increasing value of $\delta$. The FB states have a well defined spatial configuration depending on their specific propagation constant as well as on the value of $\delta$ [see the examples shown in Fig. 2b for $\delta =1$]. FB states at $E=0$ have only four amplitudes, while FB modes at $E=\pm V_2$ have six. These states require a perfect cancellation of phases at connector sites^24^ [blue circles in Fig. 2b], in order to forbid the flow of energy to the rest of the lattice. Therefore, the condition $A=-\delta B$ is mandatory for the horizontal (*A*) and vertical (*B*) amplitudes, respectively, which couple to the connector sites. We observe that the profiles become symmetric in amplitude (excepting a phase) for $\delta =1$, and that they asymmetrize for $\delta \ne 1$. The participation ratio of these compact states is given by $R_0=2(1 + \delta ^2)^2/[N(1 + \delta ^4)]$ and $R_{\pm V_2}=2 (2 + \delta ^2)^2/[N(2 + \delta ^4)]$ [see gray and red curves in Fig. 2c]. For $\delta <1$, the FB states have larger amplitudes at their sides and, therefore, they are better connected to the lattice and may allow transport. As $\delta \rightarrow 1$ the FB states become symmetric and occupy an increasing number of lattice sites: $R_0=4/N$ and $R_{\pm V_2}=6/N$. For $\delta >1$, the three FB states reduce to a profile with only two large amplitude at their center, which may favour localization. This suggests the possibility of a dynamical transition around $\delta =1$, which can be mediated by a change in coupling constants, something that could be triggered, for example, by mechanical forces^35^ or by morphological changes during learning in neuronal systems^36^.

## Numerical analysis and dynamical properties

We study numerically the alternated lattice model by exciting the dendrite at a short spine region [see green disk in Fig. 2d-inset] and by varying the parameter $\delta$. We project all lattice sites on a one-dimensional row as shown in Fig. 2d. The energy radiates through the dendrite for $\delta <1$, while it becomes localized as the spines become more compressed by increasing $\delta$. The FB states at $E=\pm V_2$ have a non-zero amplitude at the excitation site [see Fig. 2b], which increases linearly with $\delta$. Therefore, the excitation of these two FBs determines the observed localization in this case. For $\delta >1$, there is a weak radiated background coming from the dispersive bands of the spectrum. To characterize this dynamical transition, we plot in Fig. 2e the output participation ratio $R(z_{max})$ versus the compression parameter $\delta$, for short (red) and long (gray) dendritic input excitation regions. We observe how *R* decreases linearly around $\delta =1$, as an indication that a dynamical transition towards localization has occurred. The manner in which the FB states change, as spines are compressed/elongated, is related to the mode dynamics: a given site excitation will excite all the linear modes having non-zero amplitude at the excitation region (see Section S2 of Supplemental Information). Generally speaking, compression mechanisms induce an increment of the lattice conductivity due to a reduction of the lattice spacing^37^. However, contrary to this general believe, in our dendrite-inspired lattice, a compression of the lattice increases the parameter $\delta$ and generates a stronger excitation of the FB states. Therefore, the trapping of energy is enhanced in the dendrite-spine system. The increment in $\delta$ produces a more prevalent vertical (spine) interaction and, therefore, the excitation of more compact wavepackets which could be thought as energy/information reservoirs. Then, this energy can be released to the dendrite again and radiate after a new spine elongation process.

**Figure 3:**
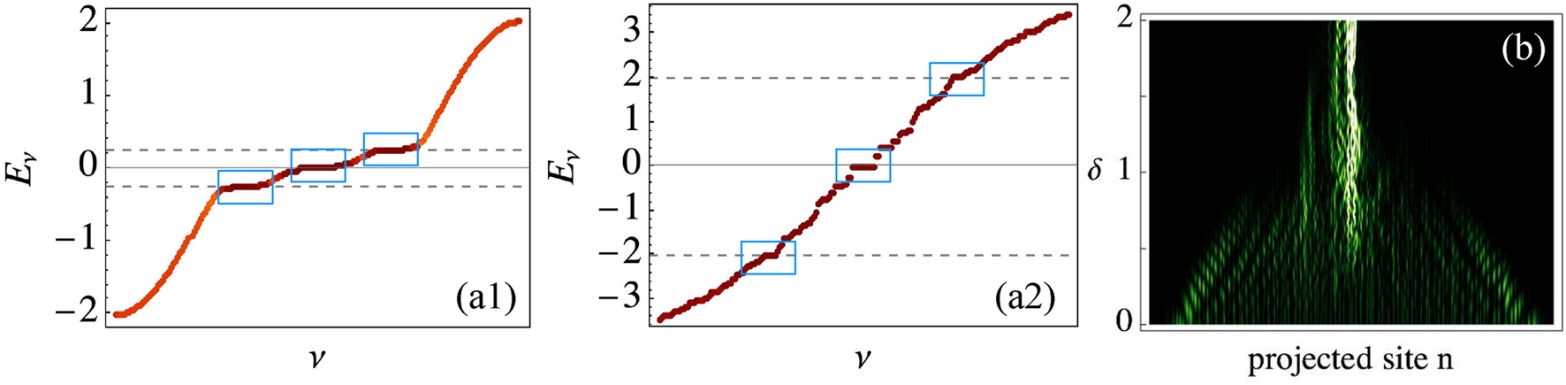
(**a1**) and (**a2**) Spectrum for a disordered lattice with $\delta =0.25$ and 2.0, respectively. Dashed lines indicate $E=\pm \delta$. (**b**) Projected output intensity profiles at $z_{max}=45$ vs. $\delta$, for a central excitation of a disordered lattice.

Interestingly, the FB transition described above is also valid for disordered systems, as the one sketched in Fig. 1d. This is because FB states exist as soon as FB units appear through the lattice, irrespective if the system is ordered or not^29^. In our case, these FB units correspond to the combination of short-long-short and long-short-long spines [see red rectangles in Fig. 1d]. We test this by studying a disordered lattice with 200 sites in the dendrite and by randomly distributing zero, short or long spines. The only condition we impose is that the spines point in the same direction every two dendritic sites, in order to avoid coupling in between spines. As soon as the system is large enough, the FB unit is repeated several times along the lattice and a FB is created independently of the lattice disorder, as shown in Fig. 3a by blue squares. We corroborate this by numerically exciting a central site in the dendrite and by varying the parameter $\delta$. Fig. 3b collects our results and shows quite clearly the dynamical transition close to $\delta =1$. Statistically speaking, the averaged $R(z_{max})$ versus $\delta$ shows a tendency similar to Fig. 2e, with two different dynamical regimes around $\delta =1$ (see Section S3 of Supplemental Information).

**Figure 4:**
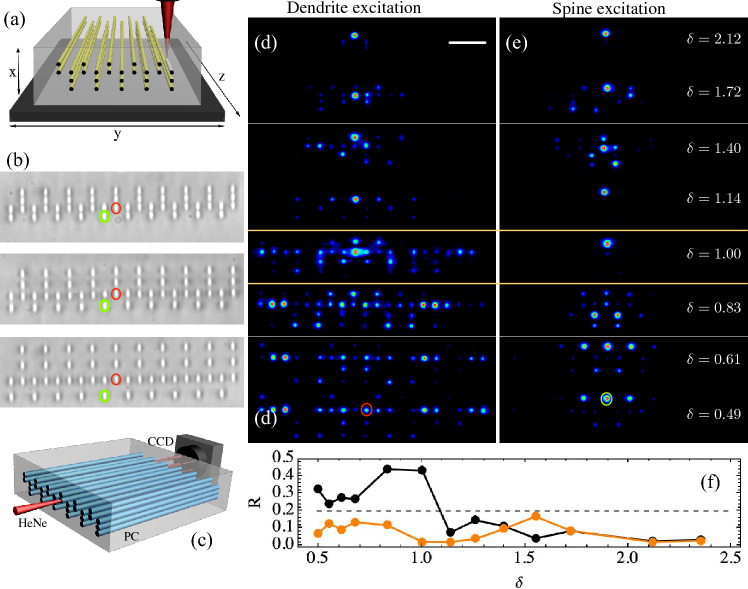
(**a**) fs laser writing technique. (**b**) Three different dendritic photonic lattices with vertical distances $b=13, 20.2,$ and $25\ \mu$m. (**c**) Characterization setup. (**d**) and (**e**) Output intensities for a dendritic and a short spine excitation. $\delta$-value is indicated directly in figure. Scale bar 50 $\mu$m in (**d**). (**f**) Participation ratio versus $\delta$ for: dendrite (black) and short spine (orange) excitations. Dashed line corresponds to $R=0.2$.

## Experimental implementation

We demonstrate our theoretical findings by means of a photonic platform and verify experimentally the different dynamical regimes predicted by our dendrite-inspired model. Photonic lattices are fabricated using a femtosecond (fs) laser writing technique^38^, as sketched in Fig. 4a (see Section S4 of Supplemental Information). The glass sample has a length of $L=5$ cm and the waveguides are fabricated along the whole glass. Due to the axial fabrication technique, our waveguides have an elliptical transversal profile^38^ of approximately $4\times 11\ \mu$m^2^. We fix the horizontal distance to $a =19\ \mu$m, and experimentally vary the delta parameter by changing the vertical distance *b* ($\delta \approx 1$ was obtained for $b=20.2\ \mu$m). In total, we fabricated 13 dendritic photonic lattices of 50 sites each with vertical distances *b* ranging from 12 to $27\ \mu$m. In this geometry, $V_1=1.04$ cm$^{-1}$ and $V_2\in \{0.5,2.5\}$ cm$^{-1}$ (see Section S5 of Supplemental Information). Fig. 4b shows bright-field microscope examples of three photonic dendritic lattices having a different distance *b*. Every lattice is characterized using the setup sketched in Fig. 4c, where a HeNe laser is tightly focused at the input facet of a photonic chip (PC) by a 10$\times$ microscope objective (not shown in the figure). The light travels along the PC and the output profile is collected using a CCD camera connected to a computer.

We experimentally excite the unit cell (see Section S6 of Supplemental Information), and study the dendritic dynamics. Fig. 4d and e show a selected sequence of output intensity images, after exciting the short spine region at the positions indicated by a red (dendrite) and green (spine-head) ellipses in Fig. 4b. For a dendrite excitation we observe that, for a small value of $\delta$, the dendrite interacts only weakly with the elongated spines, and that the energy flows preferably along the dendrite, in a spatial distribution similar to discrete diffraction^30^. However, when crossing the critical value $\delta =1$, there is an abrupt dynamical change with the energy collapsing into a reduced region. For $\delta >1$, we observe a localized oscillatory pattern which essentially corresponds to a linear combination of FB compact states (see Section S2 of Supplemental Information). This dynamical transition is in excellent agreement with our theoretical prediction and demonstrates how the compression of spines-like channels on an alternated dendritic system may induce an abrupt dynamical transition. On the other hand, for all values of $\delta$, the excitation of a short spine-head [see Fig. 4e] shows a rather constant (vertically elongated) oscillatory pattern on a very reduced lattice region, with a structure resembling the FB mode profiles. As we infer from Fig. 2b, the excitation of a short spine-head excites the three FBs simultaneously and, as a result, a compact and quasi-periodic dynamical profile is expected [see the oscillatory localized pattern in Fig. 2d for $\delta >1$]. After varying $\delta$, we do not observe transport through the dendrite, but a compactification of the energy in the region of short and long spines. We quantify the experimental data by extracting the waveguide intensity and by calculating the respective participation ratio. We plot the results in Fig. 4f for the two excitations shown in Fig. 4d and e. This quantification reveals a clear dynamical change in the energy transport for an excitation at the dendrite (black curve): transport is observed for $\delta \leqslant 1$ ($R\gtrsim 0.2$), while after crossing the critical value the energy localizes on just few lattice sites ($R\lesssim 0.2$). For a spine-head excitation (orange curve), localization is observed always for any value of $\delta$.

## Conclusions

Inspired firstly by the spatial organisation of spines on dendrites, and secondly by the compartementalization of synapses in spines coupled with dendrites, we have introduced in this work a photonic lattice configuration that allowed us to study the properties of energy transport in ordered and disordered lattices, for a wide range of parameters. We have shown that when considering lattices with alternated short and long spines, a distinctive phenomenology emerges. In this case, the localization of energy for a spine-head site excitation occurs due to the existence of FBs in the spectrum. We demonstrate a dynamical transition from transport to localization, which is controlled by the relative coupling of the spines to the dendrite chain ($\delta$), interpreted here as a compression or dilation of the lattice. This provides a mechanism for a system to transit between two dynamical states, thus acting as a gate/switch and regulating the degree of transport of energy. In physical systems, this could be achieved dynamically by a local dilation, for example, through changes in temperature or mechanic deformation, or biologically, by readjusting the morphology and/or configuration of spines by other active means^35,39,40^. We verified numerically that a disordered configuration could show a similar dynamical transition, as soon as the system is large enough to allocate several FB units. This allows us to extend our results to a more general and complex environment, where the spines are not periodically located along the dendrite.

Although we acknowledge that the focus of this work is not to provide a model for biological dendrites, a geometric mechanism as the one described here, acting in the linear regime only, may provide a simple way for multiple spines to regulate and integrate signals locally, at the level of a few spines, before transmitting them through the dendrite. However, we note that this is purely hypothetical, and more work is required to learn if such a linear mechanism is plausible in dendrites. Until now, spines have mostly been understood as isolated receptor centres that regulate the passing of electric signals to the dendrite by a high resistance neck connecting the two. In our photonic analog, the resistance would be directly proportional to the separation distance (*a* or *b* in Fig. 1) and inversely proportional to the coupling constant (see Section S5 of Supplemental Information). Open questions remain on how the nature of the coupling—for instance, evanescent or ionic—may alter the dynamics observed in the photonic lattices. Biologically, it is still unknown which are the mechanisms of electrical compartmentalization of spines, and if features of spine-dendrite coupling may or not support a dynamical transition from transport to localization. We would like to point out that our dendrite-inspired setup is a purely linear system, that shows a dynamical transition triggered by dilation or compression. This could be a fruitful new approach for bio-inspired photonic actuators and other devices, working at low energy regimes compared to more costly nonlinear systems^41^.

## Methods

### Sample fabrication

 In order to fabricate the photonic lattices used in our experiments, we use the femtosecond laser writing technique^38^. By focusing ultra short pulses from a laser beam on a borosilicate wafer, we are able to locally modify the refractive index. Then by moving the sample at fixed velocity, this change of the refractive index is obtained along the sample and creating a waveguide inside the glass plate. This procedure can be repeated at different positions creating the desire photonic lattice.

### Supplementary Information

Supplementary Information.

## Data Availability

The datasets generated during and/or analyzed during the current study are available from the corresponding author on reasonable request.
