# Supplementary material for: Transport and localization on dendrite-inspired flat band linear photonic lattices
Source: Sci Rep. 2023 Aug 11;13:13057. doi: 10.1038/s41598-023-39985-8 (PMC10421877; doi:10.1038/s41598-023-39985-8)
Supplement: Supplementary file 1 — Supplementary Information. [file 41598_2023_39985_MOESM1_ESM.pdf]

Supplementary Information for "Transport and localization on dendrite-inspired flat band photonic lattices"

Javier I. Cubillos[1,2], Diego Guzmán-Silva[1,2], Víctor Cornejo[3], Ignacio Bordeu[1], and Rodrigo A. Vicencio[1,2]
[1] *Departamento de Física, Facultad de Ciencias Físicas y Matemáticas, Universidad de Chile, Chile*
[2] *Millenium Institute for Research in Optics - MIRO and*
[3] *Neurotechnology Center, Department of Biological Sciences, Columbia University, New York, NY 10027, USA.*
(Dated: June 10, 2023)

S1. DENDRITE, SHORT AND LONG PERIODIC SPINE DISTRIBUTION

We first consider a 1D finite chain of $N_0 = 50$ sites [see Fig.1(b) of the main text], as a model of an isolated dendrite. This system has only one site per unit cell and, therefore, only one band given by

$$E = 2V_1 \cos k_x a \ ,$$

as shown in Fig. S1(a) for a finite system. The stationary solutions of a lattice define the linear spectrum and the transport properties of a given periodic system [1], which are found by using a Bloch-like ansatz where k_x corresponds to the transversal momentum along the dendrite direction and "a" to a dendrite spacing [see Fig.1(b) of the main text]. E defines the propagation constant of the lattice (normal) modes along the dynamical direction z. In this case, all the linear modes are spatially extended, with an average participation ratio of $R \approx 0.7$ [see an example as inset in Fig. S1(a)]. Therefore, when this chain is excited at any lattice position [see Fig. S1(b)], the energy simply radiates along the structure, with a standard discrete diffraction pattern, having a linear increment of R over z due to a ballistic propagation regime [1]. This is a very well known result for 1D chains, and show that a 1D lattice indeed mimics a waveguide as a system to disseminate energy. In the neuronal analogue, a 1D lattice represents a dendrite, functioning as medium for the propagation of stimuli [2].

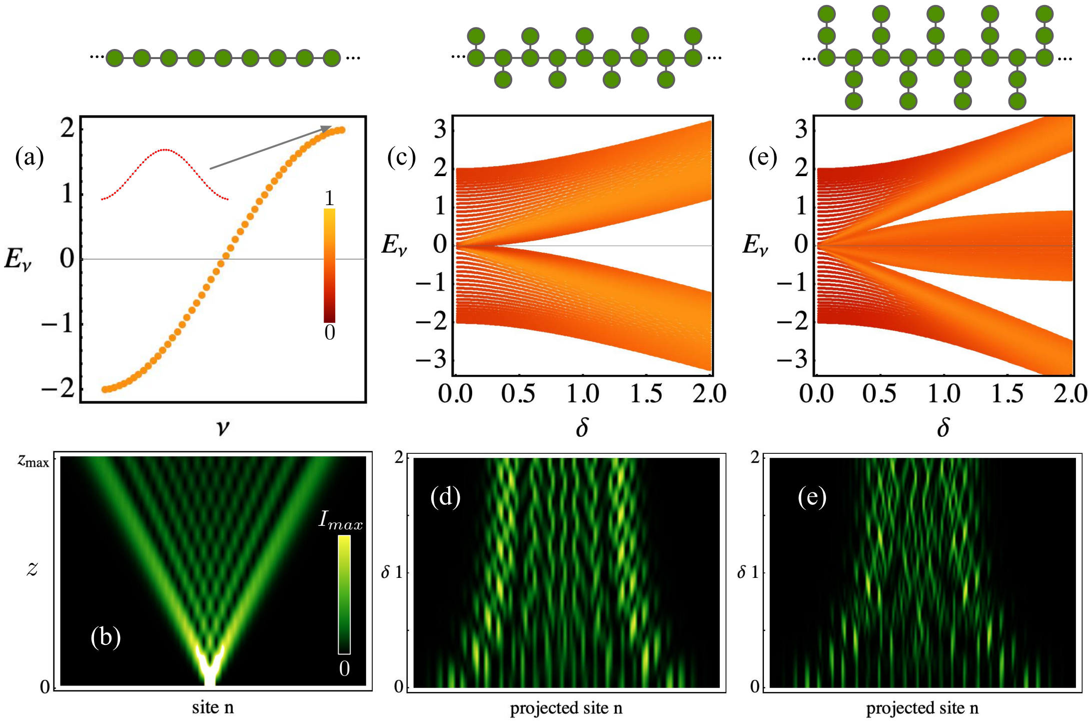

Figure S1. (a) Band spectrum versus mode index ν for a dendrite chain ($N_0 = 50$). The color indicates the participation ratio R of every mode, using the scale shown as inset in (a). (b) Discrete diffraction pattern along the propagation direction, after exciting a central site on a dendrite. (c) and (e) Band spectrum for a dendrite with short ($N_0 = 100$) and a dendrite with long ($N_0 = 150$) spines, respectively. (d) and (f) Projected output intensity profiles at $z_{max} = 10$ cm for different values of ratio δ for a dendrite with short and long spines, respectively. $V_1 = 1$ cm^{-1}

Now, to study the effect of spines in the lattice properties, we first consider a dendrite-model with regular and periodic distributions of "short" spines. We consider a configuration of alternating short-spines, forming the lattice sketched in Fig.1(c) of the main text, where the spines can not interact directly between them and do so only through the dendrite chain. This lattice has only two sites per unitary cell and, therefore, two bands, which are given by

$$E_{\pm} = V_1(\cos k_x a \pm \sqrt{\cos^2 k_x a + \delta^2}) \ ,$$

with $\delta \equiv V_2/V_1$ as a compression parameter and V_1 and V_2 corresponding to the dendrite (horizontal) and spine (vertical) coupling coefficients, respectively. We plot the band spectrum in Fig. S1(c) for a finite lattice of $N_0 = 100$ sites and for different values of δ. In this case, the modes have an average effective size in the range $R \sim \{0.34, 0.62\}$,

where all the states are extended and occupy most of the lattice. Therefore, a single-site excitation (stimuli) will propagate through the lattice, similar to a 1D dendrite chain, with only dispersive bands excited. We characterize the dynamics in this lattice by fixing the propagation distance to $z_{max} = 10/V_1$ and by varying the ratio δ. We obtain the projected output profiles $|u_n(z_{max})|^2$ shown in Fig. S1(d), where we observe a well disseminated energy pattern and that the radiated energy spatially shrinks as δ increases (for a larger scale z_{max}, the energy will cover the lattice anyway). As no energy localization is observed in this short spine lattice, no dynamical transition from transport to localization is possible in this periodical system. Similar results are obtained when considering long spines only [Fig.1(d)], where the spines spanning two lattice sites are connected to the dendrite. The unit cell in this case is composed of three sites, resulting in three nontrivial bands, which are obtained as solutions to the cubic equation

$$E^3 - V_1 \cos(k_x a) E^2 - 2V_2^2 E + 2V_1 V_2^2 \cos(k_x a) = 0 \ .$$

By computing the spectrum of a finite system with $N_0 = 150$ sites and varying the compression parameter δ, we obtain the band spectrum shown in Fig. S1(e). Similarly to the short spine case, here the linear modes are very well distributed over the lattice, with $R \sim \{0.38, 0.54\}$. In Fig. S1(f) we show the projected output dynamics $|u_n(z_{max})|^2$, as a function of δ, and observe how the energy only spreads through the system, without any possibility of transiting into localization in a linear regime [1].

S2. UNIT CELL DYNAMICAL EXCITATION

The dynamics depends exclusively of the linear spectrum in linear systems. For a given initial condition, the modes that will be excited are the ones who have an amplitud different to zero at the excitation region. In addition, for a dendritic model, we observe that the compression parameter δ also affects the spatial distribution of modes and, therefore, the excited spectrum could change while deforming the lattice. We numerically integrate the model (1) by initializing the system at a single site (delta-like excitation) and studying the dynamics over two different propagation distances, as shown in Fig. S2(a) and (b). In Figs. S2(c) we show the excited spectrum, obtained after Fourier transforming the light field along the propagation coordinate z, and integrating the information by summing over the whole lattice. In this way, we can study which frequencies and modes are excited on a given dynamical case. We observe essentially the same dynamics, but contaminated for reflected waves in (a) due to the larger propagation distance. We compute the participation ratio R for all output profiles and include an inset in each figure. We observe a clear transition into localization for a dendrite short-spine excitation [see Figs. S2(a1) and (b1)], while not that clear for a dendrite long-spine one [see Figs. S2(a3) and (b3)]. This can be understood by inspecting the excited spectrum in panel (b) and observing that for the first case the FB spectrum exceeds the dispersive one for $\delta > 1$, while for the second case the dispersive spectrum is always predominant. When exciting the spines at their heads, we observe that always the FB is larger than the dispersive one and, therefore, we observe a localized and oscillatory spatial pattern [see Figs. S2(a2), (a5), (b2) and (b5)]. The spine-long middle excitation produces a mixture spectrum with a not very clear nor abrupt transition, due to the fact that for an increasing δ parameter the spectrum is narrower and the increment in R is smooth.

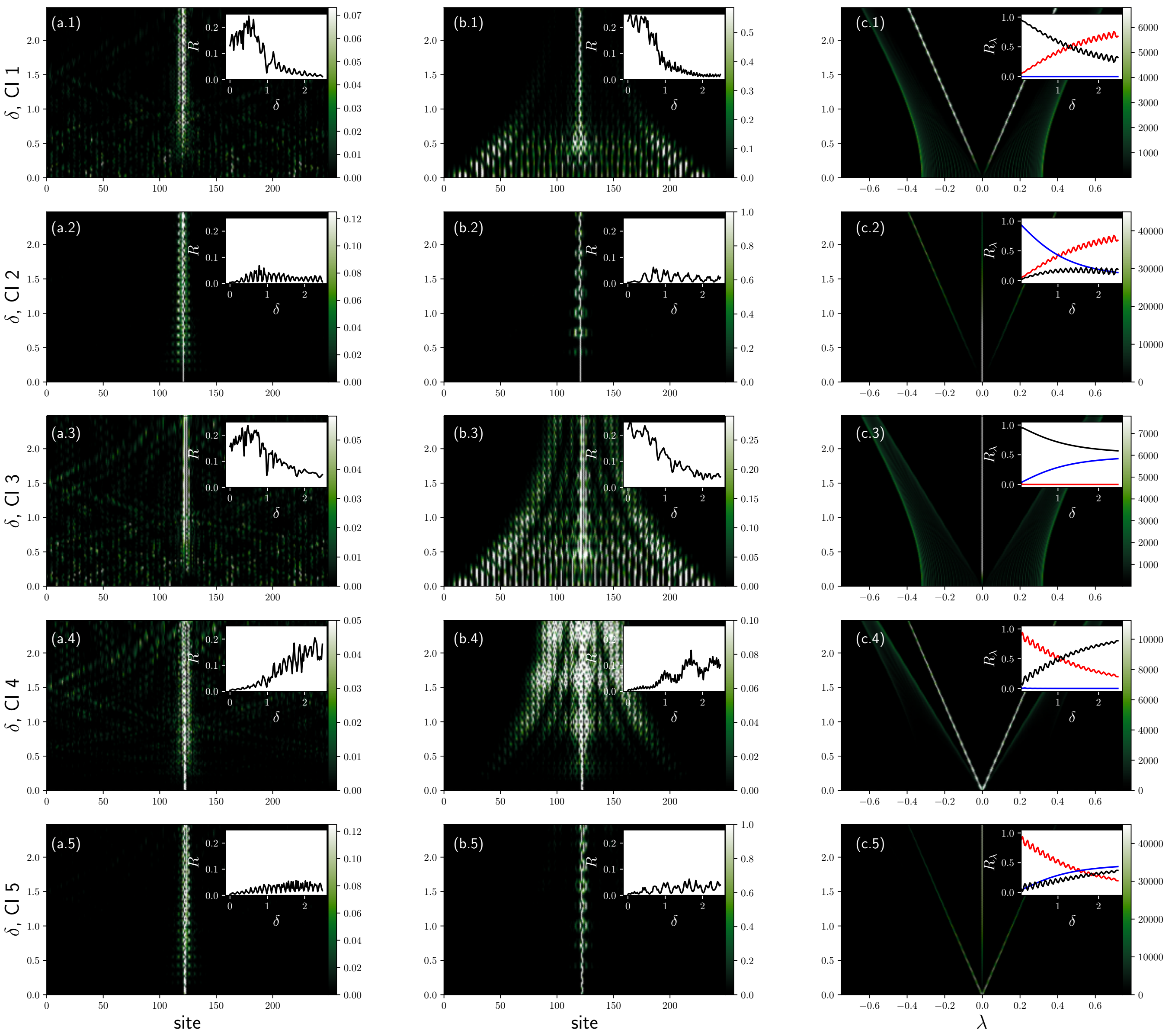

Figure S2. (a) and (b) Projected output profiles $|u_n(z_{max})|^2$ for $z_{max} = 200$ and 22, respectively, for the following input excitations: (1) dendrite short-spine, (2) spine-short, (3) dendrite spine-long, (4) spine-long middle, and (5) spine-long top. Every plot includes the participation ratio R versus the compression parameter δ. (c) Excited spectrum for (b) figures. We include a spectral participation ratio R_λ versus δ, with band differentiation: $E = \pm V_2$ in red, $E = 0$ in blue, and the dispersive bands in black. We considered a lattice with 250 sites.

S3. DISORDERED LATTICES

We study the effect of disorder in the distribution of long, short or zero spines. For this, we consider dendrite lattices with 100 sites and 50 realizations for each value of δ, as the one sketched in Fig. S3(a). We excite every lattice at a central dendrite position and characterize our results by computing the average participation ratio $\bar{R}$ at a distance $z_{max} = 22$ in the interval $\delta \in \{0, 2.5\}$. We define the randomness in every lattice by assigning a probability p_1, p_2, and p_3 for long, short, and zero spine lengths, respectively, such that $p_1 + p_2 + p_3 = 1$.

In all the cases shown in Fig. S3, we observe that two dynamical regimes are predominant. For $\delta < 1$, the average participation ratio has a value $\bar{R} \gtrsim 0.2$; i.e., the energy is well disseminated through the lattice as shown in Fig.(2) of the main text. For $\delta > 1$, $\bar{R} \lesssim 0.1$ and the energy become trapped on a small lattice region. The cases where $\bar{R}$ stays constant over δ correspond to homogeneous lattices where no localization-delocalization transition is expected.

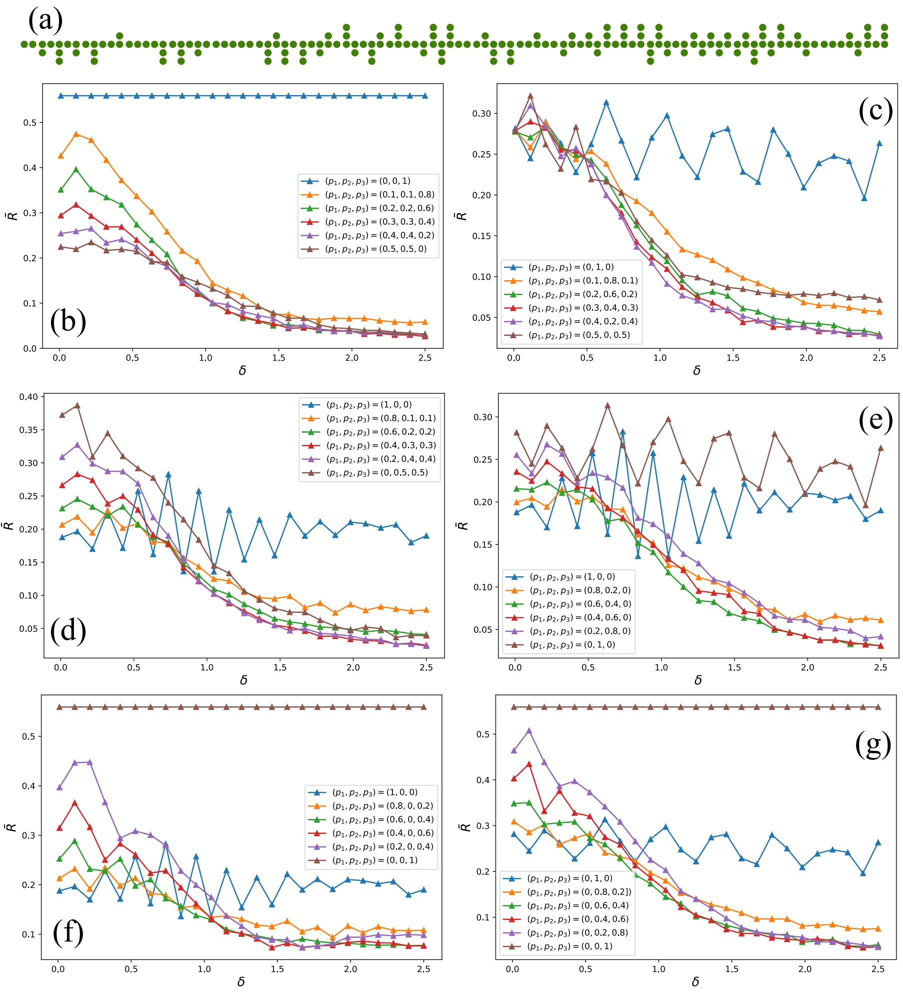

Figure S3. (a) A disordered lattice example. Averaged participation ratio $\bar{R}(z_{max})$ versus the compression parameter δ for the following cases: (b) $p_1 = p_2$, (c) $p_1 = p_3$, (d) $p_2 = p_3$, (e) $p_3 = 0$, (f) $p_2 = 0$, and (g) $p_1 = 0$.

S4. FEMTOSECOND LASER WRITING TECHNIQUE

Photonic lattices composed of optical waveguides are fabricated using a femtosecond (fs) laser writing technique [3], as sketched in Fig.3(a) of the main text. Ultra short pulses, at a wavelength of 1030 nm and pulse width of ~ 230 fs (red beam in the figure), are tightly focused inside a borosilicate Eagle XG glass wafer (gray block in the figure) using a 20× microscope objective. The glass is continuously translated with a motorized Thorlabs XYZ stage (black plate in the figure) and waveguides are created inside the sample (yellow cylinders inside the glass). The refractive index change has a contrast of $\Delta n \sim 10^{-4} - 10^{-3}$, with a nominal refractive index of $n_0 = 1.48$ for the borosilicate glass [4]. The writing velocity and power was set to $v = 0.4$ mm/s and $P \sim 80$ mW, respectively, obtaining single-mode waveguides operating at 632.8 nm (HeNe laser). The glass sample has a length of $L = 5$ cm and the waveguides are fabricated along the whole glass. Due to the axial fabrication technique, our waveguides have an elliptical transversal profile [3] of approximately 4×11 μm.

S5. COUPLING CHARACTERIZATION

We characterize the coupling constants in our lattices by using a dimer model; i.e., two waveguides separated by a given distance d. If, for example, the waveguide 1 is excited, a periodic transfer of power will occur and the power will be simply given by

$$P_1(z) = P_0 \cos^2(Vz) \;\; \text{and} \;\; P_2(z) = P_0 \sin^2(Vz) \; ,$$

where $P_0 = P_1(0)$ corresponds to the input power. The characterization method is based on this oscillatory dynamics, but considering a short evolution with less than one full cycle. The idea is to measure the transferred power from waveguide 1 to waveguide 2, when the coupling interaction is starting to occur. Fig. S4(a) shows an example of this with three different coupling constants V such that $V_1 > V_2 > V_3$. There, we observe that a smaller coupling constant implies a smaller amount of transferred power at an equal distance. Therefore, we experimentally extract coupling constants by fabricating dimer systems composed of a long waveguide 1 ($L_1 = 5$ cm) and a short waveguide 2 ($L_2 = 0.6$ cm) as sketched in Fig. S4(b). We fabricate two sets of five dimers each to characterize the horizontal

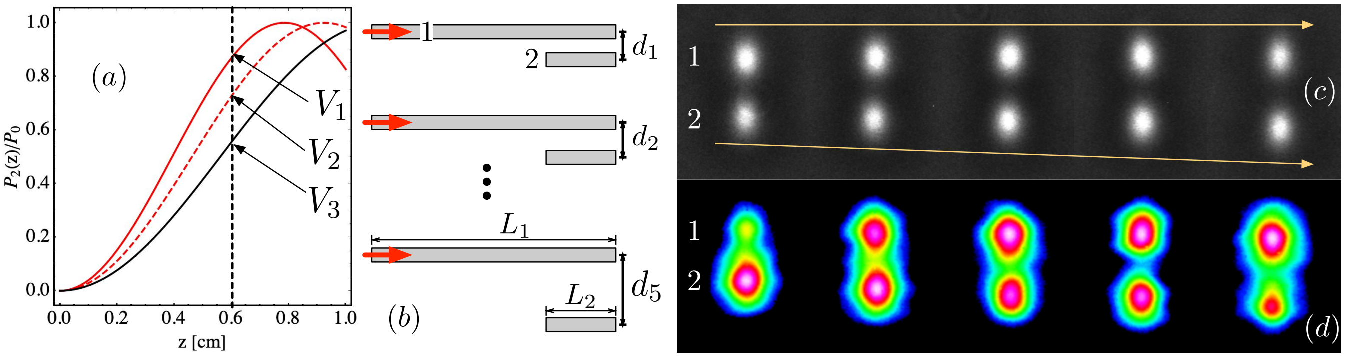

Figure S4. (a) Theoretical normalized power at waveguide 2 versus propagation distance z, for three different coupling constants as examples. The vertical dashed line indicates the propagation distance for waveguide 2. (b) Experimental scheme to characterize coupling constants over separation distance d_i, with $i = 1, 2, ..., 5$. (c) White light microscope image at the output facet of five vertical dimers with separation distances $16, 17, 18, 19, 20$ μm. (d) Output images after laser (HeNe) excitation of waveguide 1 [see red arrows in (b)].

and the vertical coupling constants. As an example, we show a white light image for the vertical case in Fig. S4(c), where d is growing to the right. Every dimer is excited by a HeNe laser excitation at waveguide 1 and output intensity images are taken with a CCD camera, as the examples shown in Fig. S4(d). We analyze these images in a gray scale and extract the information about the intensity at waveguide 1 and the intensity at waveguide 2. Then, we use power expressions to calculate the corresponding coupling constants as follows

$$\frac{P_2(L_2,d)}{P_1(L_2,d)} = \tan^2(VL_2) \;\rightarrow\; V(d) = \frac{1}{L_2}\tan^{-1}\left(\sqrt{\frac{P_2(L_2,d)}{P_1(L_2,d)}}\right) .$$

In this way, we obtain the coupling constants $V(d)$ as a function of separation distances d as shown in Fig. S5.

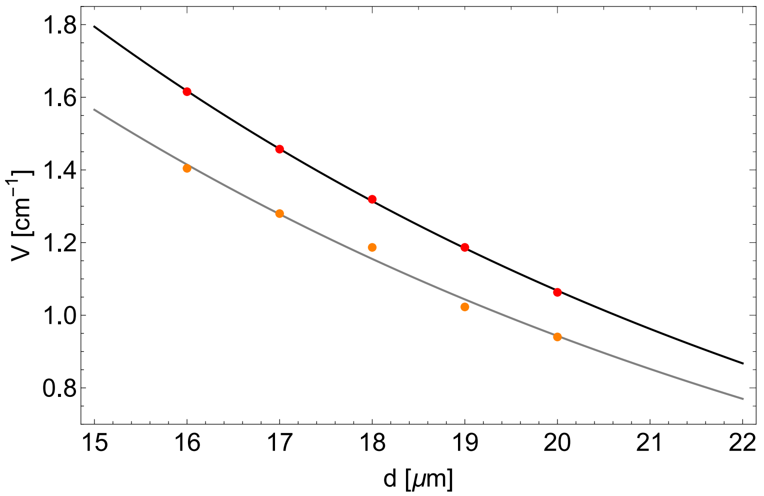

Figure S5. Experimental coupling vs distance. The orange (red) points correspond to the coupling obtained for horizontal (vertical) distances. Gray (black) line corresponds to an exponential fit for orange (red) data points.

We fit the obtained data with an exponential expression $Ae^{-\alpha d}$ and obtain

$$V_1(d) = 7.17\ e^{-0.10d} \quad \text{and} \quad V_2(d) = 8.52\ e^{-0.10d}\ ,$$

for horizontal and vertical coupling constants, respectively.

S6. UNIT CELL EXPERIMENTAL EXCITATION

We experimentally excited the dendritic lattices at the five different unit cell bulk sites using a characterization setup as the one shown in Fig.3(c) of the main text. We observe a dynamical transition close to $\delta = 1$ for a dendrite excitation (first and second columns), observing transport for $\delta < 1$ and a tendency to localization for $\delta > 1$. The excitation of the spine shows a rather compact pattern (third to fifth columns), what indicates a predominant excitation of compact FB states. In fact, it can be observed in these cases an oscillatory profile which can be as compact as a single-site profile, what indicates a strong compactification of the energy when exciting a spine.

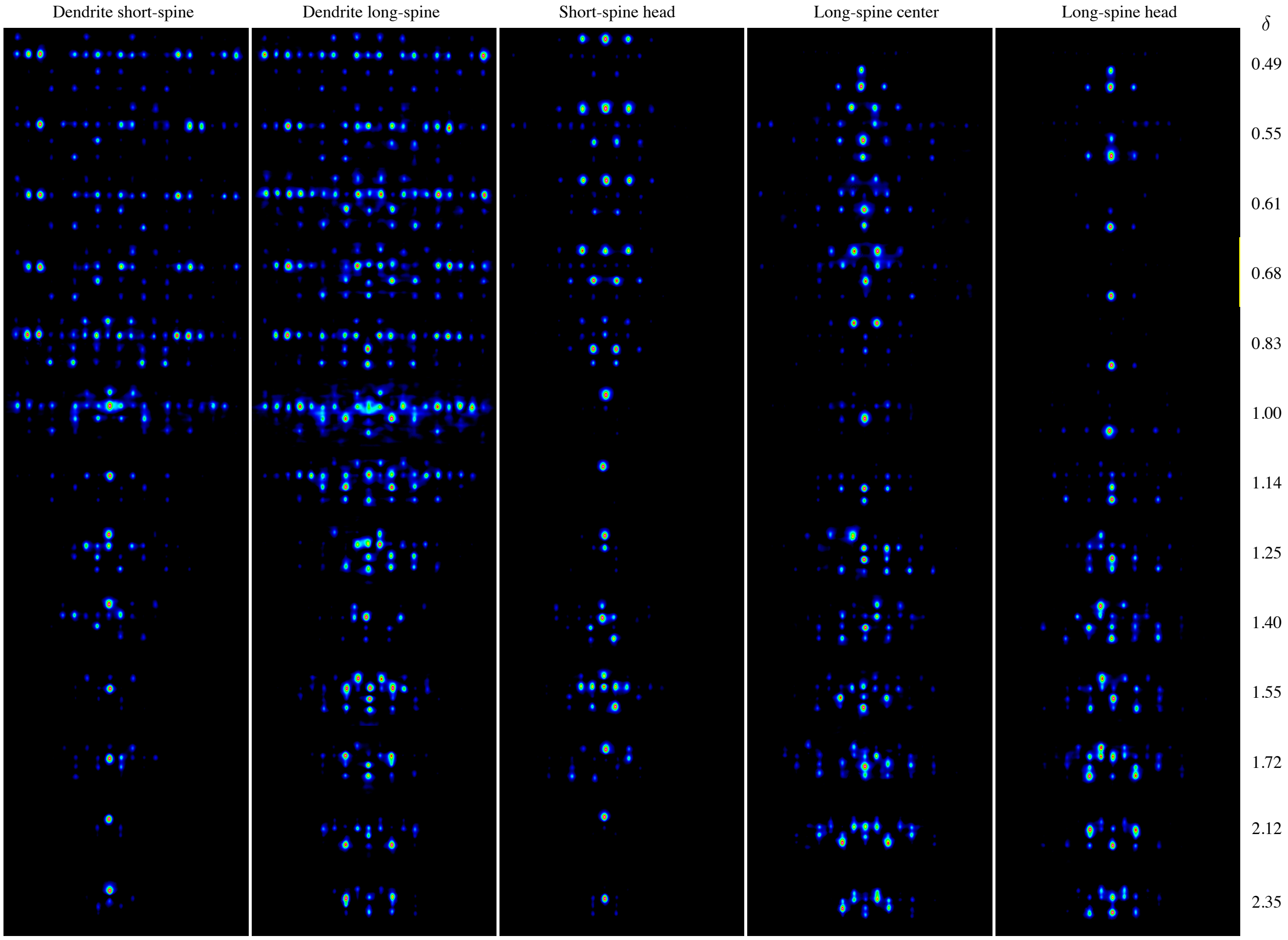

Figure S6. Experimental output images for different site excitations at the unit cell, and for different δ parameters, as indicated in the figure.

[1] F. Lederer, G. I. Stegeman, D. N. Christodoulides, G. Assanto, M. Segev, and Y. Silberberg, Phys. Rep. **463**, 1 (2008).
[2] M. Roelandse and A. Matus, J. Neurosci. **24**(36), 7843-7847 (2004).
[3] A. Szameit, D. Blömer, J. Burghoff, T. Schreiber, T. Pertsch, S. Nolte, A. Tünnermann, and F. Lederer, Opt. Express **13**, 10552 (2005).
[4] D. Guzmán-Silva, G. Cáceres-Aravena, and R. A. Vicencio, Phys. Rev. Lett. **127**, 066601 (2021).
